# Forms of explanation and why they may matter

**DOI:** 10.1186/s41235-018-0143-2

**Published:** 2018-12-29

**Authors:** Jonathan Baron

**Affiliations:** Department of Psychology, University of Pennsylvania, 3720 Walnut St., Philadelphia, 19104 PA USA

## Abstract

Explanations from neuroscience are threatening to replace those from psychology in the eyes and hands of journalists, university administrators, granting agencies, and research students. If replacement happens, much of psychology will exist only as part of the historical record. It, thus, may be useful to understand what forms of explanation are used by the two fields. Such an understanding may help us explain how each field can contribute to the other and why they are different. I review several templates of psychological and neuroscientific explanation, and criticize some others. I argue that psychology (and neuroscience) should continue to exist. Neuroscience is not better than psychology, and it cannot replace psychology.

## Significance

This article tries to explain why psychology should continue to exist in its traditional form.

## Introduction

Many people have recently been concerned, even alarmed, about the increased attention given to neuroscience in situations that would, in the past, call for psychology. These situations include accounts in news media, academic hiring in psychology departments (often now renamed to reflect the new emphasis), allocation of funds by granting agencies, stated principles for diagnosis of (what used to be called) psychological disorders, and the law.

My purpose here is not to cover all this ground but to reflect on what should count as explanations in psychology and neuroscience, thus putting in perspective the role of explanations from neuroscience. I write not as an expert on the philosophy of psychology but mostly from experience, primarily as a journal editor, but also as one who has “lived through” the last 60 years of the history of psychology and neuroscience.^1^

## Reduction to a lower level

Several philosophers, perhaps beginning with Ryle (1949) and, more to the point, Fodor (1968), have argued that concepts relevant to psychology are no less real or scientific than those relevant to biology. Moreover, reduction to a lower level of explanation is not necessarily better, just different. The principles of physics are relevant to many aspects of games played with balls or pucks. However, our knowledge that balls take a parabolic path in a vacuum, or that the path of tennis balls also depends on aerodynamic and elastic forces as well as the angular momentum of the ball, plays very little role in understanding the game of tennis. Most of the top players probably do not bother studying the relevant physics at all (although maybe they should!).^2^

High-school science courses, and basic college courses, represent fields that have demonstrated their importance over centuries, yet most of them involve very little reduction. The principles of electronics (such as *E*=*IR*), the basic laws of thermodynamics (*PV*=*nRT*), and much of introductory chemistry can ultimately be explained by quantum physics (or so I have been led to believe), but that reductive explanation plays essentially no role in the design of electronic circuits, automobile engines, or the production of wine. Modern biology relies more on reduction to chemistry, but the basic rules of genetics stand on their own, as do many of the principles derived from the theory of evolution.

Psychology, too, has plenty of principles and results that stand on their own without reduction. Examples of some of the more scientific ones are Stevens’s power law, the robustness of signal-detection theory’s rejection of high thresholds, the Rescorla–Wagner theory of learning, and the drift–diffusion model of response time and accuracy. Of course, each of these theories has limits, as do the principles we learn in basic science courses. My point is that these are scientific in every sense and are not based on reduction to biology. At a more practical level, we have such findings as the superiority of spaced over massed repetition in learning (Melton, 1970), the superiority of statistical over clinical prediction (Meehl, 1954), the relevance of phonemic analysis in learning to read English (Treiman, 1991), the overconfidence of eyewitness testimony (Bornstein & Zickafoose, 1999), confirmation bias (Nickerson, 1998), the lack of broad transfer of practice of simple skills (Ericsson, Chase, & Faloon, 1980), the role of deliberate practice (Ericsson, Krampe, & Tesch-Römer, 1993) and automatization (Bryan & Harter, 1899) in the acquisition of expert skills, the effectiveness of cognitive behavioral therapy for many disorders (Butler, Chapman, Forman, & Beck, 2006), hyperbolic discounting and its consequences (Ainslie, 1975), the curse of knowledge (epistemic egocentrism; Camerer, Loewenstein, & Weber, 1989), the mythical fixed pie in negotiation (Bazerman, Baron, & Shonk, 2001), and hundreds of other such results, some of which are ignored but should not be.

A lack of understanding of the existence of the science of psychology, and its achievements, may partially explain the recent attention to neuroscience.

## Psychological explanations

An understanding of the nature of psychological, and neuroscientific, explanations may help clarify the difference between psychology and neuroscience. As an editor, I have repeatedly had to deal with papers that seemed to me to provide explanations that should not count as psychological, or even scientific. These cases forced me to think about what would count. I have come to think that psychological explanations follow certain templates (schemata), basic forms with details to be filled in.

The following templates are tools of psychology that are used for prediction, control, and understanding of behavior and mental states. (I discuss some neuroscience templates later.) For this purpose, they must refer to states that are observable (for prediction) or manipulable (for control). The following templates account for most or all of the explanations I find acceptable (at least for cognitive psychology), but I have no way to be sure that I have not missed something big. These templates do not vie with each other in the way that competing theories sometimes do; they may coexist and continue to be useful for different purposes. Historically, however, many of them have arisen in response to difficulties that other templates had in producing convincing accounts of certain phenomena.

In listing these templates, I want to make three points. First, psychology is a limited field, defined by the types of explanations it provides. Other fields of inquiry, such as economics and sociology, are also concerned with human behavior and experiences, but they have different tools for explanation (with some overlap). Second, these templates *work*. They succeed in providing explanations that are useful for prediction, control, or understanding. Third, they are scientific. Explanations in psychology may be tested, criticized, improved, and even rejected. In sum, psychology is a science, like many others. It does not need to be replaced by neuroscience, just as chemistry does not need to be replaced by physics.

***Association***

The oldest template may be the idea of association, which was apparent in the writings of philosophers as early as Aristotle. Simple associations may be seen as a network of connections among psychological objects of some sort, with each connection characterized fully by two objects and a strength. Usually the objects were mental representations. Carl Jung, following many 19th century psychologists, used a word-association test as a way to probe the contents of minds, and the idea of association was central to Freud’s theory of dreams, slips of the tongue, and hysterical symptoms.

The basic idea is that two objects are associated as a result of experience, perceived similarity, or some innate connection. When one object is later stimulated in some way, it evokes the other. Stimulation may result from the presentation of a stimulus, and the effect may be observed through its effect on some response.

A modification of the basic idea was to assume that there are different kinds of associations. For words, these might include super-ordinates, sub-ordinates, co-ordinates, complements, synonyms, opposites, and so on. People could limit reported associations to one type or the other. The idea of labeled connections between nodes potentially increases the capacity for representation, so long as the use of a network of associations was not limited to spreading activation from node to node, determined only by the strength of their connections. Thus, the idea of association could provide some basis for other templates to be described.

The simpler idea of association has been an essential component of explanations of memory since the beginning. It is still very much alive in psychology, particularly in such effects as semantic priming (Schvaneveldt & Meyer, 1973) and in priming studies that fill the journals. For example, the call to prayer is associated with prosocial behavior (Duhaime, 2015). Often such studies rely on what might be considered very weak associations involving chains rather than direct connections. The results are often difficult to replicate. However, the basic idea of semantic priming has been repeatedly confirmed.

***Stimulus–organism–response: additive models***

For Pavlov and others, the associations of interest were not (just) between mental representations but also those between stimuli and responses. Pavlov inspired more sophisticated theories of learning based on the idea, such as that of Hull (1943). The Rescorla–Wagner (1972) theory of learning builds on these earlier theories and goes beyond them, still retaining stimulus–response and stimulus–stimulus associations as primary tools of explanation. Much of this work and theorizing was on the conditions under which such associations were formed. It was assumed that many of them (Pavlov’s reflexes) were innate.

Along the way, people started to think of simple stimulus–response associations, with nothing in between, as insufficient. The classic textbook by Woodworth and Schlossberg (1954; I read the 1960 revision for my introductory psychology class) notes that the organism also needs to be considered, so its standard model was S-O-R. (They used “organism” rather than “subject” because they were already using S for stimuli!) The basic framework here is still that of association, but with two steps: that between stimuli and some internal representation of it, which is usually also affected by characteristics (temporary or long-lasting) of the organism, and that between the internal representation and its expression in a response. Moreover, the internal representations can combine inputs from two or more stimuli into a single representation.

Brunswik used such an approach in studies of perception, which led to the early work of Kenneth Hammond (Dhami & Mumpower, 2018). The contemporary study of judgment—in which a subject must produce a quantitative evaluative response when presented with a multi-attribute stimulus—is full of explanations of this type. The common idea was that cues (features or attributes) of stimuli in the world were first represented internally, then weighed and added up to produce a perception or judgment.^3^

One slightly extended application of this approach is found in the work of Michael Birnbaum and others who study quantitative judgments in which the cues are also quantitative (e.g., Birnbaum & Veit, 1974). The general idea is that in a judgment task, two monotonic transformations occur. One of these is between a stimulus and its internal representation. Internal representations are then added with weights, or subtracted (for judgments of differences), to produce a summary representation, which is then transformed by another function into a response, mapping a feeling of intensity to (e.g.) a number. The response-producing function can differ for different responses, even if they come from the same internal representations, thus producing scale convergence.

A related approach is conjoint measurement, in which internal representations are inferred from comparative responses (e.g., Krantz, Luce, Suppes & Tversky, 1971; Tversky, 1967). In sum, a standard form of psychological explanation accounts for the connection between stimuli and responses in terms of weighted additive combinations of (internal representations of) cues in the stimuli.

***Goal-directed systems***

In part as a reaction to the Pavlovian tradition, some psychologists (e.g., Irwin, 1971; Skinner, 1938) started to emphasize a different type of explanation, often attributed to Thorndike (1911), who proposed the law of effect to account for the effect of reward on the frequency of behavior leading to the reward (analogous to the role of reproductive success in the theory of evolution). We can explain behavior by its consequences. Resistance to this idea came from the feeling that this was not a casual explanation, since causes of behavior must precede the behavior. However, this was not really a problem because the general approach obviously required some sort of prior learning or some other reason for an animal to represent the connection between behavior and its consequences.

This approach found full expression in the work of the (self-described) behaviorist B. F. Skinner, but the general approach is not limited to behaviorist explanation. It can be extended to cognitive explanations, in which choices are explained in terms of beliefs and desires. Beliefs can be affected by learning but can arise in other ways. Desires or goals include those resulting from drives, but these too can arise in other ways. The general idea is that we explain S’s choice of C by showing that “S chooses C because S believes that C will more likely lead to outcome O, which S prefers to alternative outcomes.” The slots that need to be filled in are those concerning beliefs and desires/goals.

This sort of explanation is common in the study of decision-making, which also refers to a normative standard, a tool for evaluating decisions as better or worse, in which beliefs are represented as probabilities of particular outcomes, and desires for outcomes are represented as their utilities (Baron, 2008). This kind of normative model is used in other fields, for example, cost-effectiveness analysis in medicine. It is sometimes used as a descriptive model in economics.

***Information processing***

Starting perhaps with Wiener’s *Cybernetics* (1948), an approach developed that explained behavior in terms of the flow of information. As computers became more powerful in the 1950s and 1960s, this approach became common in cognitive psychology, with several variants. One variant involved modeling thought with computer programs. Other variants involved flow diagrams, which could be seen as extensions of the S-O-R approach described above, with more boxes in the middle between S and R, and with the boxes doing more computing than could easily be understood in terms of networks of associations, addition, and subtraction. Still other versions of this approach involved mathematical models of the uptake and use of information.

Information processing encountered at least two sources of resistance. One was to the idea that a model was an explanation. There is something to be said for this objection. An explanation should increase understanding, but a model can (in principle) be accurate yet beyond understanding itself. Most models are understandable. That said, I occasionally see papers where the model is mathematical, a bunch of equations, and, while the model “works,” examination of the equations yields no understanding of *why* it works, and therefore no clear predictions about where else it would work aside from the data presented. It might as well be a black box with a person inside who, in fact, acts like a person, hence “fits the data” from what other people do in the same situations.

The second early objection came from behaviorism. Behaviorists objected to the postulation of internal representations because they were not directly observable. Although it might appear that observations of the brain may provide evidence of those internal states, it is difficult (as I argue later) to identify brain events with the postulated internal states in an information-processing model. Ericsson and Simon (1980) argued (convincingly to most cognitive psychologists) that, with some care, verbal reports could serve as evidence of the postulated states.

In information-processing models, the slots that need to be filled in are essentially the same ones needed for a description of a computer algorithm, that is, a flow chart. Each step requires inputs in the form of some representations, and an output, which may serve as an input to another step. In addition, the step must be described in terms of a function that produces outputs from the inputs. Importantly, some steps may require conditional decisions: “If this, do that.” Such flow charts (sometimes described only verbally) have been used extensively in the study of heuristics for decision-making and many other areas.

### What makes a good template?

Successful templates work because they correspond to reality, at least in cognitive psychology. We cannot observe associations directly, just as we cannot observe electrons.^4^ However, in some sense, we know that they exist. We can create them and then show in many ways that they have been created. Similarly, people (and probably many other animals) often make decisions by considering the possible outcomes and the relative value that they have, as well as their certainty of occurring. Moreover, the flow of information in tasks such as reading can be broken down and observed piece by piece. These templates are not in themselves theories or hypotheses, but each one provides a language and set of assumptions, within which theories may be stated and questions may be asked. For example: Are associations symmetric? Does information flow in one direction only or is there feedback from higher (later) levels to lower ones? Which functions operate in parallel and which in series? The corresponding questions can be answered in ways that allow prediction, control, and understanding.^5^

A second property of a good template is that it is adaptable to applied psychology and the questions that it poses. If we want to influence people’s decisions, the simplest way (not always possible) is to change their values for outcomes or their beliefs about which outcomes are likely to occur as a result of each option. In education, we can create useful associations and avoid creating harmful ones. If we know how information is processed, we can discover where errors are generated and thus, find ways to reduce them. Conditioned responses (of Pavlov’s sort) have been part of behavior therapy since the outset.

An example is the field called judgment and decision-making, which is based on a design consisting of three models (Baron 2012): normative, prescriptive, and descriptive. Normative models specify standard by which judgments and decisions are evaluated. Sometimes they are just “distance from the right answer,” for example, in the case of numerical judgments of quantity, but they may also involve probability theory or expected utility theory. Descriptive models attempt to explain how people do the tasks in question, with special attention to the relation between their responses and the normative model. Prescriptive models are plans for applications. Based on the results of normative and descriptive models, they are designed to promote judgments that are closer to the normative model. The descriptive models are within psychology, but clearly the concepts that they employ must make some reference to the terms of the normative models, such as subjective probability or utility, and they must also refer to objects that can function in prescriptive models.

The concepts of subjective probability and utility are imposed on the situation, much like longitude and clock time. The earth did not come with north–south stripes on it, and the mind (or brain, for that matter) does not come with a probability meter. Yet we can study how probability judgments are related to relative frequencies, whether they are consistent with each other, and how probabilities can be best communicated. We have, in fact, learned quite a bit about these issues. Likewise, in the study of decision-making under risk, for example, extensive analysis has been done of the various ways people make use of information about value and probability information in combination.

Some templates are banging the door to be let in to the list of standard ones (as others have done in the past). One that I think should not be admitted involves the use of metaphor. A comparison of metaphor with more useful templates might help to elucidate their advantages.

An example of the use of metaphor is construal level theory (Trope & Liberman, 2010), *as it is applied* by many researchers.^6^ The idea is that thinking processes can be characterized as high or low in their level. The high/low dimension corresponds metaphorically to several others: abstract/concrete, distant/nearby (in time, space, or social distance), and holistic/detailed. It is often applied to situations where the interpretation is less obvious, for example, by claiming that one attribute of a stimulus is higher than another (when the attributes could be things like price and quality, or dollars and probability). A typical experiment might involve manipulating or measuring one of these attributes and looking for a relation with another. For example, an increase in perceived distance might lead to more holistic processing.

It may be possible to make sense of several parts of this account. For example, representations that are distant are likely to be less detailed. However, such an account would not explain other inferences from the theory, such as the effect of level (high/low) on the discount rate, or on holistic vs. analytic. Which of these is higher? Analytic thinking may be more detailed (lower), but it may also be more cognitively advanced (hence, higher). Abstract thinking may be higher than concrete thinking, but we sometimes speak of deep thought, which would be lower.

Moreover, it is unclear why manipulation of perceived distance should affect the type of thinking that is done. The associations of dimensions are not assumed to operate through the usual association mechanisms (discussed earlier). It is not as if the word “future” evokes the concept “abstract,” which then, in turn, causes the subject to think abstractly. The idea that “the dimensions are associated one way because most people see them that way” does not seem to me to answer the question of why temporal distance increases abstract thought, if it does. Metaphors may lead to predictions, but it is hard to see how they lead to understanding.

## The proper place for neuroscience

### Neuroscience templates

Neuroscience has its own templates for explanation. Here are some of the more traditional ones.

One is very much like the information-processing models described above. The explanation concerns the flow of information through the nervous system. However, the fundamental concepts are somewhat different. The major descriptors of these circuit diagrams are inhibition, excitation, and modulation (i.e., modifying the strength of an inhibitory or excitatory link). Conditional, “if then,” nodes (of the sort used in information-processing models) are not fundamental and may even be difficult to explain. Moreover, the inputs and outputs to each transition are identifiable groups of nerve cells and synapses, not representations of the sort used in information-processing models.

Elaborations of this template concern how connections are strengthened and weakened as a result of past inputs. These principles are meant to account for learning and forgetting, and they may do that, but the descriptions are in terms of neural inputs rather than psychologically meaningful representations. Of course, in some cases the relation between neural inputs and psychological representations are well understood (e.g., many sensory and motor systems).

Another sort of template concerns localization of function. Although many sophisticated methods are now used to determine localization, much was already known by the 1950s—before the widespread use of microelectrodes for this purpose—about the human brain based on studies of brain lesions such as strokes. The general form of the explanation is a statement about some area of the brain (or peripheral nervous system) and some psychological function that it serves. This knowledge has a different purpose from that of psychology, being more closely related to applications of neuroscience in neurology and neurosurgery. The descriptions of psychological functions of anatomically definable sets of nerve cells may, but need not, correspond to elements of the psychological templates described above, which were designed for different purposes.

An example of a standard neuroscience flow explanation is the neuroscience of visual perception, which has been traced through the retina, the lateral geniculate body, a few rings of the visual cortex, and to the superior temporal lobe of various mammals, including humans. Cells at successive levels respond to increasingly abstract features of visual stimuli. Experience using connections is needed to maintain them, and connections may be inhibited (suppressed) by some sort of modulation (as occurs in strabismus-induced amblyopia, an interesting condition that I have myself). Although we are increasingly sure that complex visual recognition, such as your recognition of the words of this text, occurs in the temporal lobe, somehow, the search for grandmother cells has not yet completely succeeded.

### The role of neuroscience in psychology

In many cases, it may be possible, eventually, to map the neural circuit diagram into an information-processing flow diagram, node for node, thus providing a full reduction of the psychological explanation to neuroscience, or, we might say, vice versa. Moreover, such a mapping might facilitate the design of medical interventions, such as drugs for treating a psychological disturbance (or electrical brain stimulation for similar purposes). However, it would probably not help much in the design of interventions that can be understood in terms of psychology alone, such as corrective lenses and hearing aids. Even the treatment of strabismus amblyopia—requiring toddlers to wear a patch over the good eye—can be understood in terms of perceptual learning (plus the concept of critical periods to explain why it works best with toddlers), although, of course, our understanding of why the treatment works (when the toddler cooperates, which I did not) would be improved. From outside the head, the concept of perceptual learning and the concept of strengthening neural connections through use are equivalent.

Moreover, even a full neuroscientific understanding of visual perception will not go far in explaining complex skills, such as reading, that involve the visual system. Yet the psychology of reading, as an exemplary skill that has been studied since the 19th century (Huey, 1908), is fairly well understood. (I would say “very well understood” up to the part that involves understanding language, where the same problems arise as with understanding spoken language.) It is not at all clear how a richer understanding of neural circuits could improve on the understanding we have achieved based on psychology alone. At best, we could say that the information-processing flow diagram is parallel to a neural circuit. This is nice to know, but it is not a replacement for the flow diagram. For psychology, it does nothing that the flow diagram cannot already do.

The localization template is more difficult to translate meaningfully into any psychological template. In particular, studies of localization of function need not describe functions in ways that can be translated into psychologically meaningful elements. Examples are in the frontal lobe of the cortex [especially the dorsolateral prefrontal area, DLPFC; see Greene (2013), Chapter 4, and the current Wikipedia article], which has been described as involved in self-control, cognitive control, inhibition of impulses, utilitarian reasoning (vs. deontology), reason (vs. emotion), working memory, planning, and abstract reasoning. It may turn out that all these functions reduce to a single, theoretically meaningful, psychological function, such as a tendency to inhibit automatic responses, but that remains to be demonstrated. In the meantime, it is simply unclear how to map what the DLPFC does in any direct way into concepts of the sort studied in psychology. It is not clear what psychology has to gain, for psychology alone, by asking whether it can tie together all the functions of the DLPFC, or any other part of the frontal lobe, with a single theory.

I do not see how neuroscience could plausibly be a drop-in replacement for psychology, just as physics is not a drop-in replacement for chemistry. The terms of the two approaches do not map neatly into each other. Moreover, if they did for some part of each field, we would still have no reason to banish either of the two approaches. The parts that match would be redundant, to be sure, but each of these parts would fit into a larger whole that lacks this redundancy.

That said, neuroscience can provide explanations that are outside of psychology, when psychology, limited to its standard templates, fails. Examples abound in the psychology of motivation, emotion, sensation, and perception. Drives such as hunger, thirst, sex, and (the need for) sleep are fairly well understood in terms of their underlying physiology, even though much can also be understood based on psychology alone (e.g., Premack, 1959, and subsequent follow-up work). The psychological basis of color vision was understood from psychology alone (e.g., Hurvich & Jameson, 1957), but the question “Why does it work this way?” was answered by studies of the role of the retina and the visual system of the brain. The same was true of binocular depth perception. Moreover, the dual systems involved in auditory pitch perception were explained in terms of the dual role of the cochlea, representing pitch in terms of pulses or in terms of location of stimulation. Note that these examples do not require the replacement of psychology with neuroscience but, rather, the use of neuroscience to answer questions that arise in psychology itself.

In these cases, neuroscience provides an answer that cannot be provided at all by the various forms of psychological explanation listed above. More generally, a science on its own can go only so far in answering questions, and it at some point must declare “That’s just how it is,” or, better “That’s for someone else to explain.” High-school physics (as I learned it) treated Ohm’s law (*E*=*IR*) and the ideal gas law (*PV*=*nRT*) as fundamental. There was some discussion of electrons moving through matter, and molecules bouncing around and hitting each other, but a deeper understanding of why these formulas seemed to work would require another level of explanation, such as quantum theory or statistical mechanics. I cannot think of an example from psychology in which such simple fundamental principles (such as Stevens’s power law and the Rescorla–Wagner theory of learning) have been explained with any precision by reduction.

When neuroscience is advanced as an explanation, it is most useful when it describes a physiological basis that explains a psychological finding. A clear example, again, is color vision, which is limited to three dimensions as a natural consequence of the fact that the retina has three types of color receptors. Neuroscience is needed when the psychology templates I have listed above are not sufficient to account for some phenomenon.

More generally, we can put neuroscience explanations in a category that might be called *external* explanations. They are outside the standard list of explanations that define psychology as a scientific field. Other sources of external explanations in psychology are those fields concerned with culture, including language, law, and educational practices. For example, it has been argued that failing to accept the logical task (a refusal to accept hypothetical statements, e.g., those beginning with “if”) in the study of logic (Henle, 1962) declines with a child’s age in large part because of instruction in arithmetic, in which children must accept hypothetical statements such as: “If John has 3 pencils and gets 2 more, how many will he have?” (Scribner, 1977); it is not appropriate to say, “I don’t know because I don’t know John.”

It might be argued that findings from neuroscience can suggest new avenues of inquiry for psychology. For example, the discovery that the discounting of time delay is represented in two different places in the brain might lead to a search for distinct psychological processes that affect discounting in different ways. Surely this sort of thing happens occasionally. Suggestions can come from many other places (such as observation in the real world) too. However, this suggestion effect does not justify any claims that neuroscience is somehow better than psychology.

### Methods of neuroscience: Lesions, measures, and brain activity

Different methods of neuroscience have different relations to psychology. Here I discuss two of them.

Brain lesions (including supposedly temporary lesions produced by magnetic or electrical stimulation) can often tell us about localization. If you reduce the function of some part of the brain and some psychologically measurable process changes in a meaningful way, we can conclude that the relevant part of the brain is necessary for the process.

Likewise, studies of the effects of brain lesions on various functions can tell us which functions can be separated from other functions, and which functions are more closely related (e.g., Luria, 1960). Studies of patients with lesions are like studies of the correlates of individual differences in other pathologies, or in personality traits. If a lesion (even a temporary one) knocks out one function but leaves another one intact, we have clear evidence that the latter does not depend on the former. This is also true of some measures of individual differences: if these correlate with one function but not another, we have the same sort of evidence. [For example, measures of mental ability seem to be correlated with final responses to some tasks but not with initial, intuitive responses, as found by Szaszi, Palfi, Szollosi, Kieslich & Aczel (2018).]

Sometimes, evidence from neuroscience is used to answer psychological questions by measuring various outcomes. For example, the intensity of an emotional response can often be assessed with physiological measures, such as skin resistance and heart rate, in situations where it is inconvenient or questionable simply to ask people to rate the intensity of their emotions. Such uses of neuroscience are not explanations at all. They are tools of measurement, with the same status as response-time measurement, eye-movement tracking, or introspective reports.

Studies of localized brain activity are less conclusive than studies of lesions for two reasons. First, as with lesions, the functions of a brain region need not map neatly into a classification of psychological functions. Although activity measures (including peripheral measures such as skin resistance) can tell us about features of processes, such as their intensity, we still cannot identify them with particular processes of psychological interest. For example, we cannot use skin resistance to identify emotions, only their intensity (when other influences are controlled). Each area of the brain may respond to more than one kind of activity, so we cannot directly infer from its activity the psychological process of interest. Moreover, of course, some psychological functions may occur in many different locations in the brain at once.

Second, what brain activity tells us may be psychologically irrelevant. For example, suppose that we could measure how the brain responds to information about the delay and amount of rewards (as done by Kable & Glimcher, 2007) and that (contrary to fact, apparently) one brain region responds more to amount and another region responds more to delay. This would suggest that there are two systems for making this trade-off between delay and magnitude, one preferring immediate but smaller rewards, relative to the other. Yet, it may turn out that these two systems are not psychologically separable in any way. For predicting and controlling behavior—without actually manipulating the brain itself^7^—this apparent difference may be useless. Of course, the same problem would exist if the two regions did not differ: we could not infer from this that two separate systems did not exist. They could exist concurrently in both regions, and be subject to manipulation by psychological interventions.

In addition, brain activity as usually studied is likely to be a sign, not a code (Uttal, 1967). A code is a measure of a necessary step. A sign is a measure of a side effect, which is not necessary. An example is the evoked potential to a visual stimulus, which seems to be emitted when the stimulus is perceived but does not vary with many relevant aspects of the perception itself. Signs can provide hints about where to look for explanations, but they cannot establish those explanations with much credibility.

The neuroscientific study of moral judgment (reviewed by Greene, 2013, Chapter 4) provides another example, particularly concerning the two trolley problems. In one version, the choice is whether to switch a runaway trolley heading for five people to another track where it would be heading for one. In the footbridge version, the choice is whether to push a man off a bridge, thus stopping the trolley, to save the five. Most subjects say “yes” to the first case and “no” to the second. Brain regions responsible for self-control are associated with “yes” responses to the first, and regions associated with emotion are associated with “no” responses to the second. These findings are consistent with a psychological theory in which an initial emotional response may be corrected by a subsequent reflective response, thus controlling the impulse to respond based on emotion.

However, the evidence about brain regions certainly does not establish this conclusion. It is unclear, for example, whether the actual experience of emotion occurs in people who read far-fetched stories about trolleys. The emotional regions of the brain may be involved in the cognitive representation of the “as if” emotion (in which the subject identifies the emotion that would be felt if the case were real), as well as in the emotional experience itself. In this particular case, the two-systems account has behavioral implications, which may be (and have been) tested in other ways.

## Conclusion

I have argued that psychology is a science with its own forms of explanation. These standard explanations have had considerable success. Moreover, there is no straightforward translation between the templates of psychology and those of neuroscience, or vice versa.

As in other sciences, some types of psychological explanation are more successful than others. Moreover, as in other sciences except for physics, explanation in terms of still other sciences is possible and sometimes useful. Such external explanations can answer questions that arise in the basic form of the science. However, they are not substitutes for this form, and not better when a basic explanation is available.

Failure to understand the status of psychology may lead to harmful conclusions, such as those in which granting agencies eliminate funding for behavioral research, replacing it with funding for neuroscience, because neuroscience may succeed where psychology has so far failed. For example, we cannot understand psychopathology from neuroscience, since the definition of the forms of pathology depends on their psychological symptoms. These sorts of misunderstandings are exacerbated by journalists, often helped by neuroscientists themselves, who regard a finding that something happens in the brain as true science.^8^

We could understand the neuroscience of the mind perfectly yet not be able to understand the basic phenomena addressed by psychology. We could develop a model of a brain in a computer, which would behave just like real human brains. To understand why it does what it does, we would need a psychology of our computer program.

When psychology reaches the limits of what it can explain, it looks for explanations from other scientific disciplines, just as other sciences do. Such external explanations are important for a full understanding, but they are not replacements for the original science itself. They cannot replace the original explanations because they have a different form and a different purpose.

## Footnotes

1 Before high school, I was interested in both fields, and I studied both fields through college and two years of graduate school.

2 The philosophy of reductionism is extensive and very abstract. In this section, I am trying to stick to the issues as they appear to real researchers in the relevant fields.

3 An extension of this approach goes beyond the idea of a weighted sum, replacing it with various simplifying heuristics, which are properly considered as part of the information-processing template. In this case, the information-processing approach remedied a deficiency in an earlier template.

4 Steve Link told me about the following exchange in a seminar at Stanford in the 1960s, as I recall it. Karl Pribram: “I’ve been studying the brain for a long time, and I’ve never seen one of these ‘associations’ that you keep talking about.” William Estes: “Well, Karl, they are awfully small.”

5 Computer simulations may allow prediction and control in the absence of understanding, since the function of the program itself may need to be understood.

6 It is papers from these researchers that I deal with as editor, not the original papers that defined the theory.

7 This qualification is important if we are interested in neurology rather than psychology.

8 It is still nice to know that Aristotle was wrong to think that the brain’s main function is to cool the blood.
